# Rupture of the Uterus during Labor

**Published:** 1895-04

**Authors:** L. M. Bossi

**Affiliations:** Geneva


RUPTURE OF THE UTERUS DURING LABOR.—L. M. Bossi, of Genoa, (*Nouvelle Archives d'Obstetrique et de Gynecologie*, No. 7, 1893), describes two cases of rupture of the uterus during labor. Both cases occurred in multiparæ with narrow pelves, the fetus presenting in the one case by the shoulder; in the other there was placenta previa, and the rupture took place during version by the feet. The first case, in which the child was already in the pelvic cavity and was operated on in bed under favorable circumstances, terminated favorably; the second case died.

Upon the experience of these cases Bossi concludes that the Porro operation, with dropping of the pedicle into the pelvic cavity after previous ligature with an elastic ligature, offers the best chance to end the operation quickly and safely. He rejects the suture of the uterus, as it requires too much time and in deep jagged tears is often difficult to make.

WHEN SHOULD WE OPERATE ON UTERINE FIBROIDS?—There was a time, within the memory of living gynæcologists, when uterine fibroids were regarded as wholly benign and almost sure to cease growing, if not to diminish in size, after the menopause. Contemporaneous with these views of twenty-five or more years ago, the mortality of abdominal hysterectomy was eighty per cent. These considerations had an important bearing on the question of when to operate for uterine fibroids. Now that the mortality has been reduced to one or two per cent., and it is known that uterine fibroids frequently cause the death of the patient either from repeated hæmorrhage, exhaustion, pressure, renal complications, malignant or necrotic pegeneration of the fibroid itself, and that oftentimes the tumors do not cease growing with the menopause, etc., the views regarding early operations have materially changed. Martin, of Berlin, records one hundred and ninety-six cases of fibromata in which thirty-eight were found to have undergone retrograde changes. In two hundred and five cases of extirpation of myomatous uteri, Martin found nine cases which showed carcinomatous, and six cases sarcomatous, degeneration. Leopold claims that fibromata may become fibrosarcomata, and in one of his cases he observed carcinomatous formation within the myoma. Erendorfer holds that the mucose of a fibroid uterus may become carcinomatus. Emmet states that in several instances under his observation fibroids underwent sarcomatous metamorphosis. Professor Klebs and Sir James Y. Simpson mention several similar cases. I would advise the removal of uterine fibromata whenever they cause any of the following symptoms: 1. Severe menorrhagia or metrorrhagia. 2. Severe pain from pressure. 3. Repeated attacks of pelvic peritonitis. 4. Malignant or necrotic degeneration. 5. Size of tumor so large as to interfere with the patient's movements and usefulness. 6. Cystitis, dysuria, hydronephrosis from pressure on the ureters, severe hæmorrhoids, varicosities of the lower extremities, uncontrollable reflex nervous and nutritive disturbances. 7. Repeated miscarriages, tubal and extra-uterine pregnancies, and where the tumor seriously complicates the labor. In these cases I believe operation is imperative, whether the tumor be the size of a walnut or as large as a fœtal head. To allow these symptoms to continue from month to month and from year to year, in the vain hope that the menopause will bring about a desirable result, is merely to reduce your patient's strength and remove her chances of recovery. To wait for a fibroid to grow to the size of a child's head before its removal may mean the death of your patient, either before or immediately after the operation. Dr. Irish records, in the *American Journal of Gynæcology and Obstetrics* for December, 1894, ninety-four cases of fibroids, in which forty-three of them "developed dangerous and formidable symptoms, in patients between the ages of forty-two and fifty." Calcerous, necrotic, pus-forming, cystic, sarcomatous and carcinomatous degeneration may occur while waiting for the *vis medicatrix naturæ*. Abdominal surgeons are agreed that the dangers of the operation increase *pari passu* with the size of the tumor, the age of the patient, the reduction of her vitality, and the nutritive disturbances, etc., and that fatal cases are usually the neglected ones. Repeated severe hæmorrhages, attacks of inflammation and adhesions, salpingitis, ovarian degeneration, incarceration of the tumor in the pelvis, pressure on the ureters, hydronephrosis, extra-uterine pregnancy, degeneration of the tumor, or malignant newformation, are symptoms and complications that are apt to arise at any time; nevertheless, as Dr. Cushing, of Boston, said in a recent article: "Grim and lamentable cases still occur too often where, either from timidity on the part of the patient, or from bad advice and mistaken ideas on the part of her medical adviser, the time for favorable operation has passed by. The chances of recovery have been cruelly thrown away by miserable delay and worse than useless treatment, until the patient is delivered to the surgeon sinking under her burden, a subject for the hazardous and gruesome operation, and likely then to die, leaving grief to the friends, blame to the surgeon, and disgrace to the profession."—Winslow Anderson, M. D., in the *Medical Record*.

---

Reports from New York show, that notwithstanding the hard times, there has been little falling off in attendance on the Polyclinic. The profession are not inclined to give up the exceptional advantages that are to be received from this magnificent institution.

